# Comparative Evaluation of Engineered Polypeptide Scaffolds in HER2-Targeting Magnetic Nanocarrier Delivery

**DOI:** 10.1021/acsomega.1c01811

**Published:** 2021-06-10

**Authors:** Victoria O. Shipunova, Olga A. Kolesnikova, Polina A. Kotelnikova, Vladislav D. Soloviev, Anton A. Popov, Galina M. Proshkina, Maxim P. Nikitin, Sergey M. Deyev

**Affiliations:** † Shemyakin−Ovchinnikov Institute of Bioorganic Chemistry, Russian Academy of Sciences, 16/10 Miklukho-Maklaya Street, Moscow 117997, Russia; ‡ Moscow Institute of Physics and Technology, 9 Institutskiy per., Dolgoprudny 141701, Russia; § MEPhI (Moscow Engineering Physics Institute), Institute of Engineering Physics for Biomedicine (PhysBio), 31 Kashirskoe Shosse, Moscow 115409, Russia; ∥ Sirius University of Science and Technology, 1 Olympic Avenue, Sochi 354340, Russia

## Abstract

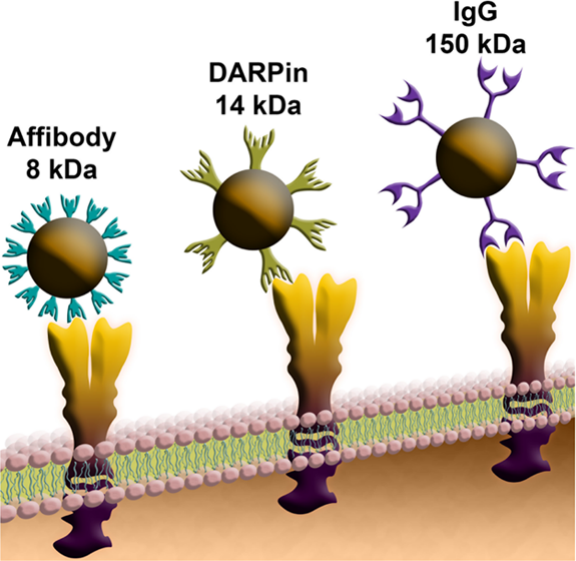

Targeted drug delivery is one of the most intriguing and challenging issues in modern biomedicine. For active targeting, full-size IgG molecules (150 kDa) are usually used. Recent studies have revealed that small artificial polypeptide scaffolds such as DARPins (14 kDa) and affibodies (8 kDa) are much more promising tools for drug delivery due to their small size, artificial nature, low immunogenicity, and many other properties. However, there is no comparative information on the targeting abilities of scaffold polypeptides, which should be taken into account when developing drug delivery systems (DDSs). The present work is the first comprehensive study on the comparison of the effectiveness of different HER2-targeting proteins within the architecture of nanoparticles. Namely, we synthesized trimodal nanoparticles: magnetic, fluorescent, and directed toward HER2 oncomarker on cancer cells. The magnetic particles (MPs) were covalently modified with (i) full-size IgG, 150 kDa, (ii) DARPin_G3, 14 kDa, and (iii) affibody Z_HER2:342_, 8 kDa. We showed that the number of DARPin_G3 and affibody Z_HER2:342_ molecules conjugated to the nanoparticle surface are 10 and 40 times higher, respectively, than the corresponding value for trastuzumab. Using the methods of magnetic particle quantification (MPQ)-cytometry and confocal microscopy, we showed that all types of the obtained magnetic conjugates specifically labeled HER2-overexpressing cells. Namely, we demonstrated that particle binding to HER2-positive cells is 1113 ± 39 fg/cell for MP*trastuzumab, 1431 ± 186 fg/cell for MP*Z_HER2:342_, and 625±21 fg/cell for MP*DARPin_G3, which are 2.77, 2.75, and 2.30 times higher than the corresponding values for control HER2-negative cells. Thus, we showed that the smallest HER2-recognizing polypeptide affibody Z_HER2:342_ is more effective in terms of specificity and selectivity in nanoparticle-mediated cell labeling.

## Introduction

Despite the significant progress achieved in cancer treatment in recent decades, the diagnostics and therapy of aggressive tumors still remain a serious problem.^1^ The most commonly used method of cancer treatment is surgery, which is often not effective for metastatic tumors. Radio- and chemotherapy are actively used as well but have a number of undesirable side effects, such as cardiotoxicity, hepatotoxicity, and many others.^2^

The rapidly developing area of nanotechnology offers the design of new effective tools for the diagnostics and therapy of cancer diseases. In particular, a promising approach is the use of highly specific nanoscale agents for the treatment without any invasive interventions. Currently, there are a number of clinically approved drugs for diagnostic and therapeutic applications, such as Ferucarbotran or Ferumoxtran magnetic agents for magnetic resonance imaging (MRI)-contrasting, or liposomal doxorubicin Caelyx for breast cancer therapy.^3^ However, despite the promise that the use of drug nanoformulations increases the solubility of drugs and reduces their administrated doses, the issues of systemic side effects of such medications are still open.

The reduction of side effects is achieved via the development of various methods aimed at increasing the therapeutic index of drugs^4−7^ and, in particular, through targeted delivery of compounds directly to the disease site. For the targeted delivery of nanocomplexes *in vitro* and *in vivo*, different proteins and peptides of various nature are traditionally used, such as antibodies and their derivatives (transferrin, epidermal growth factor (EGF), and different lectins), DNA/RNA-based molecules (aptamers and protein–nucleic acids), and small molecules (folic acid, sugars, etc.).^8−10^

Often, the use of traditional tools for targeted delivery leads to a whole range of undesirable effects. For example, the large size of full-size antibodies (150 kDa) often does not allow reaching the required number of molecules on the surface of nanostructures during chemical modification; immunoglobulin heavy chain constant domains have effector functions that can induce phagocytosis without participating in the selective target recognition, or lead to undesirable immunomodulation *in vivo*.

In the past 2 decades, targeted artificial polypeptide scaffolds of nonimmunoglobulin nature, which are obtained by phage, cell, or ribosomal display technologies, have appeared to be much more effective tools for the delivery of nanostructures to cells.^11,12^ These polypeptides, obtained by mutagenesis from protein motifs involved in protein–protein interactions in living systems, are presented by more than 20 types of non-Ig scaffolds (DARPins, affibodies, affimers, OBodies, etc.). Some of these artificial scaffolds, in particular, DARPins (derivatives of the Drosophila cytoskeleton protein, ankyrin) and affibodies (derivatives of the highly stable domain B of staphylococcal protein A) represent an excellent alternative to full-length antibodies.

DARPins are based on the natural protein ankyrin, which consists of repeating motifs of 33 amino acids. DARPins usually contain 4–6 ankyrin repeats, which are presented by 2 α-helices and a β-turn, the surface of which allows interaction with the target. The molecular weight of such scaffold polypeptides depends on the number of repeats and usually ∼14–18 kDa. The affibody molecule is based on the Z-domain of the *Staphylococcus aureus* protein A, which contains 58 amino acid residues; the Z-domain consists of three α-helices that form a barrel. There are no disulfide bridges in the structure of either DARPin or affibody. It should be noted that affibody molecules withstand high temperatures (about 90 °C) and acidic and alkaline conditions (pH from 2.5 to 11), which expands the possibilities of using this class of proteins to very severe conditions like acidic microenvironment in the stomach. It should be noted that a number of scaffold-based drugs are already undergoing clinical trials,^13^ which confirms their effectiveness in clinical practice.

These molecules are widely used for a number of reasons: small size (8–20 kDa) in comparison to full-length antibodies (150 kDa), high affinity for molecular targets (from sub-nanomolar to femtomolar constants), low immunogenicity, exceptional thermodynamic stability, and lack of cysteines in the structure. It is also worth noting the ease of large-scale biotechnological production, in contrast to full-size antibodies. These properties make it easy to perform genetic engineering manipulations and create multispecific fusion proteins that allow not only targeting nanoparticles to cells with a given molecular profile but also implementing their own diagnostic and therapeutic functions.

This work is the first comprehensive study demonstrating the effectiveness of scaffold proteins, DARPins, and affibodies for targeted delivery of nanoparticles to cells *in vitro* in comparison to full-size antibodies. In particular, colloidally stable magnetic nanoparticles coated with carboxymethyl-dextran (CMD) were synthesized by the microemulsion method. These nanoparticles were chemically modified with antibody, affibody, and DARPin directed toward receptor HER2 on the surface of cancer cells. Human epidermal growth factor receptor 2 (HER2) is a widely known oncomarker that is overexpressed in 30% of human breast carcinomas. We showed that all types of the obtained magnetic conjugates specifically and effectively label HER2-overexpressing cells. However, the small HER2-recognizing polypeptides are more effective in terms of specificity and selectivity in cell labeling, with significant superiority in affibody-modified nanoparticles.

## Results

### Synthesis and Characterization of 20 nm Magnetic Nanoparticles Coated with Carboxymethyl-Dextran

Magnetic nanoparticles (MPs) were synthesized by the water-in-oil microemulsion method. Sodium dodecyl sulfate (SDS) was used as a surfactant, *n*-butanol as an auxiliary surfactant, and *n*-hexane as an oil phase for Fe^2+^ and Fe^3+^ ions in ammonia solution. The details of the synthesis are described in the Materials and Methods section.

**Figure 1:**
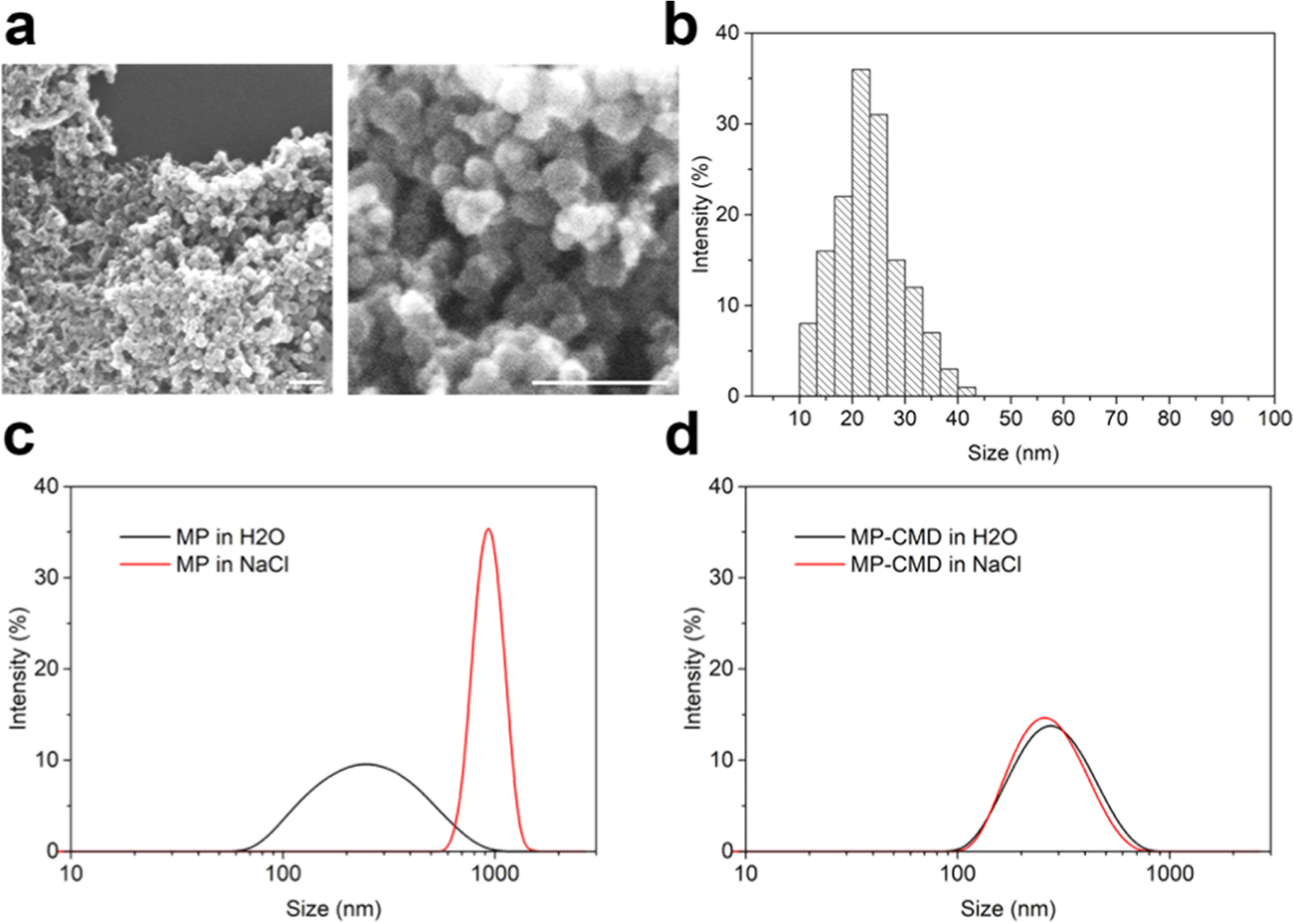
Magnetic nanoparticle characteristics. (a) Scanning electron microscopy micrographs of MPs. Scale bar, 100 nm. (b) Particle size distribution obtained by SEM image processing. (c) Hydrodynamic particle size distribution by intensities obtained for MPs in H_2_O and 1 M NaCl. (d) Hydrodynamic particle size distribution by intensities obtained for MP-CMD in H_2_O and 1 M NaCl.

The morphology of as-synthesized magnetic nanoparticles was studied using scanning electron microscopy (SEM). Micrographs of magnetic nanoparticles were obtained using a scanning electron microscope and are presented in Figure 1a. According to the results of SEM image processing, the average size of magnetite nanoparticles was found to be 23.3 ± 6.4 nm; the particle size distribution is shown in Figure 1b.

The hydrodynamic particle size distribution by intensities showed that the synthesized particles are not stable in saline solution (1 M NaCl) (Figure 1c).

Surface coating of the nanoparticles with carboxymethyl-dextran (CMD) resulted in the production of core–shell nanoparticles that are stable in saline solutions, which is shown in Figure 1d. The hydrodynamic particle size was characterized by hydrodynamic light scattering using a Zetasizer Nano ZS analyzer (Malvern Instruments Ltd.). The polymer-coated particles were found to be stable in 1 M NaCl saline solution, thus indicating the stability of coated particles under much more soft physiological conditions (usually near 0.15 M NaCl). The measurement results indicate that the hydrodynamic size of magnetic particles stabilized by a CMD polymer coating is 264 ± 113 nm and differs from the average hydrodynamic size of uncoated particles of 223 ± 111 nm, thus indicating the successful modification of the nanoparticle surface.

### Magnetic Nanoparticle Modification with HER2-Recognizing Scaffold Polypeptides

**Figure 2:**
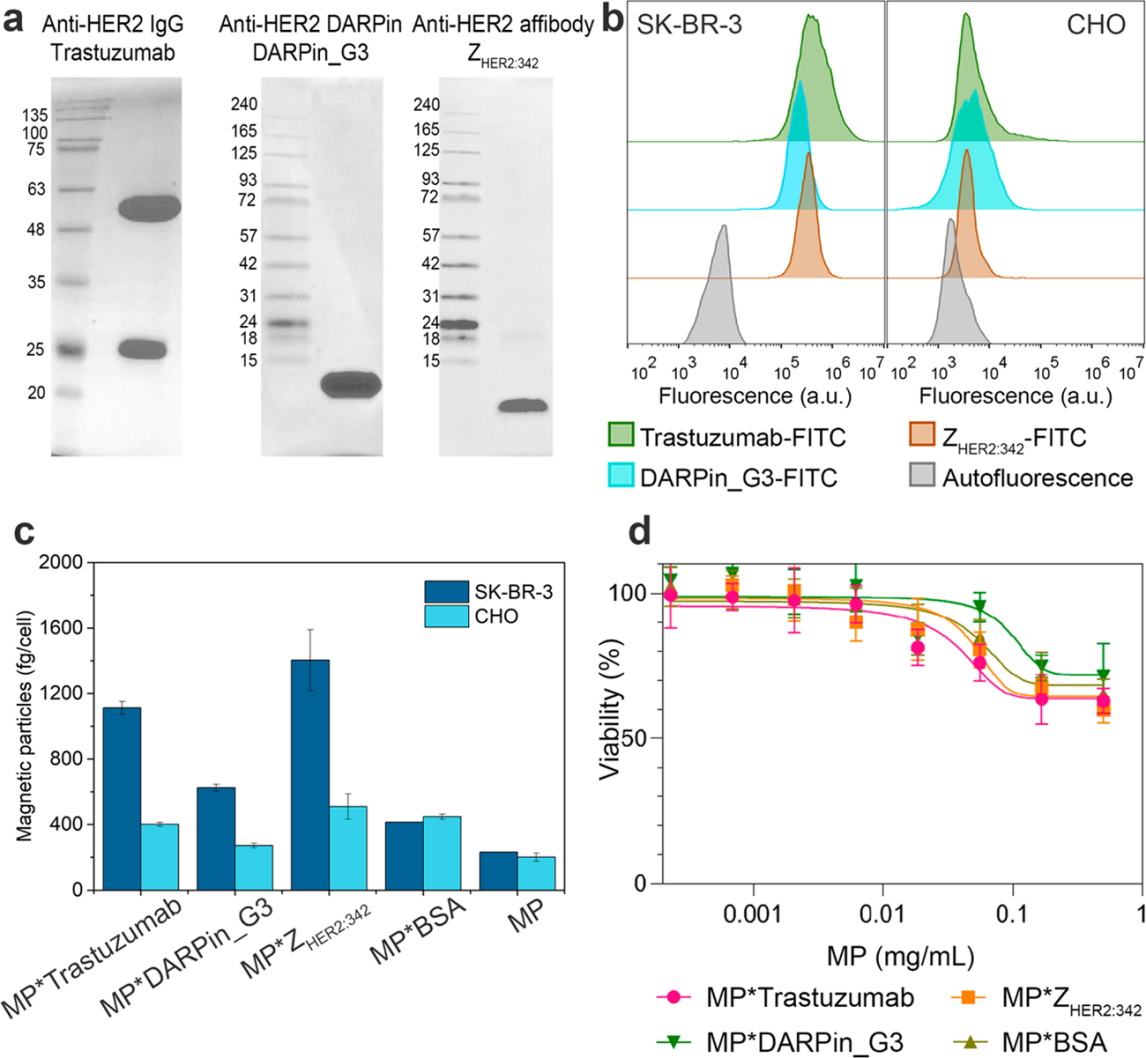
HER2-overexpressing cell targeting. (a) 15% SDS-PAGE (Laemmli) of trastuzumab (two bands correspond to the heavy and light chain of the antibody), and 10% SDS-PAGE (Tris-tricine) of DARPin_G3 and Z_HER2:342_. Numbers indicate the molecular weights in kDa of protein ladder in the corresponding conditions. (b) Flow cytometry assay on cell labeling with DARPin_G3-FITC, Z_HER2:342_-FITC, and trastuzumab-FITC. (c) Magnetic particle quantification (MPQ)-cytometry assay on magnetic nanoparticle binding to HER2-positive SK-BR-3 and HER2-negative CHO cells. Cells were incubated with MP*trastuzumab, MP*DARPin_G3, MP*Z_HER2:342_, MP*BSA, and pristine MP, washed from unbound nanoparticles and analyzed with the MPQ-cytometry device. Data presented are mean ± s.d. (*n* = 3). (d) Results on cell viability obtained with resazurin-based cytotoxicity test for magnetic particle conjugates.

As-synthesized CMD-coated magnetic particles were conjugated to protein molecules that specifically recognize the HER2 receptor. First, we used full-size, clinically approved IgG trastuzumab, which binds to the domain IV of HER2 receptor with *K*_d_ = 560 pM.^14^ Second, we used affibody Z_HER2:342_, which binds to the junction of domains III and IV on HER2 with a dissociation constant of 22 pM.^15,16^ Finally, as a DARPin representative, we chose DARPin_G3, which binds to the domain IV of HER2 with *K*_d_ = 91 pM.^17−19^ Affibody Z_HER2:342_ and DARPin_G3 were produced in *Escherichia coli* and purified with nickel columns by immobilized metal affinity chromatography via the His_6_ on both proteins. The trastuzumab was purified from stabilizing agents from commercially available medicine Herceptin (Roche). The identity and purity of proteins were confirmed by SDS-polyacrylamide gel electrophoresis (PAGE) (as described in the Materials and Methods section); the results are presented in Figure 2a.

Magnetic particles were conjugated to these three proteins using carbodiimide chemistry with EDC/sulfo-NHS (1-ethyl-3-(3-dimethyl aminopropyl)carbodiimide/N-hydroxysuccinimide) as zero-length cross-linkers. For control experiments, we used nanoparticles conjugated with bovine serum albumin (BSA). The conjugation reaction was performed as described in detail in the Materials and Methods section. On measuring the quantity of protein on the nanoparticle surface, we found that (41 ± 3) × 10^–12^ mol of trastuzumab, (433 ± 4) × 10^–12^ mol of DARPin_G3, and (1644 ± 30) × 10^–12^ mol of affibody Z_HER2:342_ were bound to 1 mg of magnetic nanoparticles.

### HER-Overexpressing Cell Targeting with Magnetic Particle Conjugates

The obtained conjugates of magnetic particles with trastuzumab, affibody Z_HER2:342_, and DARPin_G3 were used for HER2-overexpressing cell labeling *in vitro*. To this aim, we used two cell lines, namely, human breast adenocarcinoma cells SK-BR-3 and Chinese hamster ovary cells CHO. SK-BR-3 cells overexpress HER2 receptors (about 10^6^ receptors per cell), while the CHO cells do not express any EGFR receptors (family of epidermal growth factor receptors).

The expression of the HER2 receptor on SK-BR-3 cells, in contrast to CHO cells, was first confirmed with the flow cytometry assay. To this aim, we prepared trastuzumab labeled with fluorescein isothiocyanate (FITC) to get trastuzumab-FITC. Cells were incubated with trastuzumab-FITC and analyzed with flow cytometry. Histograms presented in Figure 2b confirm the overexpression of HER2 on SK-BR-3 cells, thus making use of the selected cell lines eligible. Along with fluorescently labeled IgG, we prepared fluorescently labeled affibody Z_HER2:342_-FITC and DARPin_G3-FITC to prove their HER2 binding efficiency. Both fluorescent proteins were used for the flow cytometry assay. Data presented in Figure 2b confirm that all of these proteins are equally suitable for targeting HER2-overexpressing cells with similar labeling efficiency and without nonspecific binding.

Cells were labeled with all types of magnetic conjugates with recognizing proteins, washed from nonbound conjugates, and analyzed with our original MPQ-cytometry method (magnetic particle quantification cytometry).^20^ The detection principle of this method is based on quantitative analyses of nonlinear superparamagnetics in small volumes without any background signals from linear dia- and paramagnetics such as cells or plastic.

The MPQ-cytometry assay showed that all obtained conjugates possessed specificity in terms of labeling of HER2-overexpressing cells. Namely, we showed that the binding to SK-BR-3 cells is 1113 ± 39 fg/cell for MP*trastuzumab, 1431 ± 186 fg/cell for MP*Z_HER2:342_, and 625 ± 21 fg/cell for MP*DARPin_G3, which are 2.77, 2.75, and 2.30 times higher than the corresponding values for CHO cells. It should be also noted that the conjugates of nanoparticles with BSA and pristine unconjugated particles exhibited significantly lower binding to both types of cells, SK-BR-3 and CHO. The efficiency of this binding was comparable to the nonspecific binding of the targeted conjugates to the control CHO cells. The corresponding data are presented in Figure 2c. Thus, we obtained HER2-specific conjugates of magnetic particles with proteins of different origins.

It should be noted that the designed nanoparticles have practically no cytotoxicity according to the results of a resazurin-based cytotoxicity test (Figure 2d). Namely, the calculated values of IC50 were found to be 1.08 g/L for MP*trastuzumab, 1.12 g/L for MP*Z_HER2:342_, 1.25 g/L for MP*DARPin_G3, and 1.13 g/L for MP*BSA.

**Figure 3:**
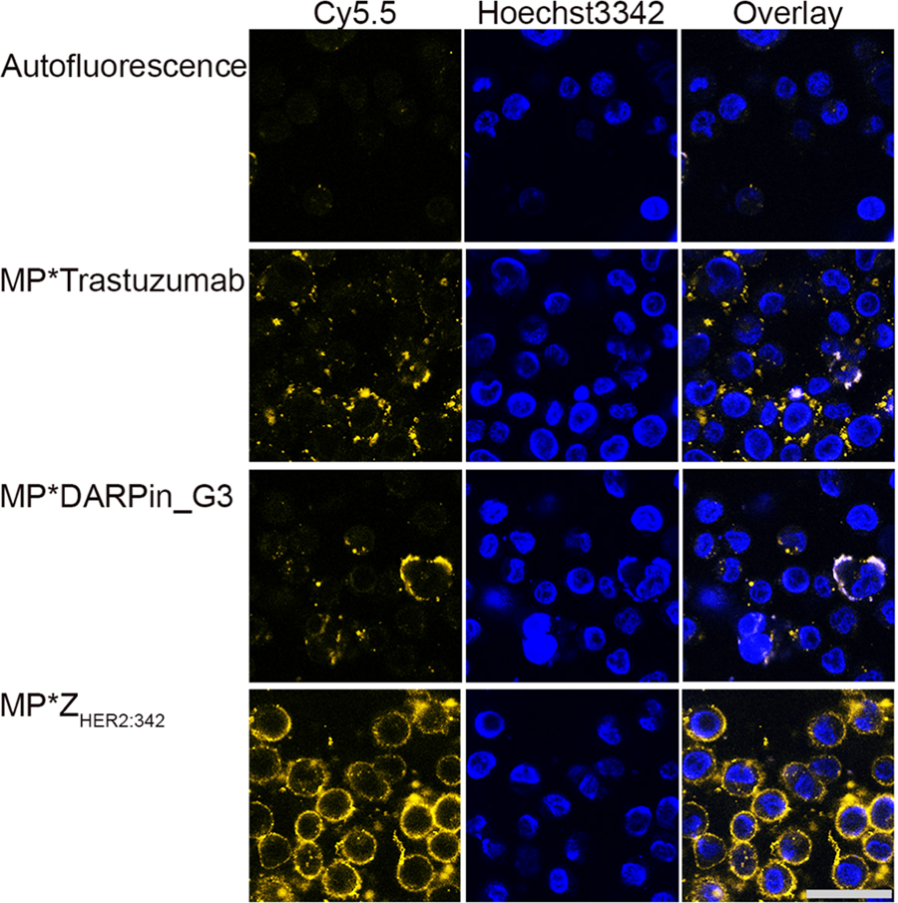
Confocal laser scanning microscopy of cells labeled with Cy5.5-modified conjugates of MP*trastuzumab, MP*Z_HER2:342_, and MP*DARPin_G3. Left: images in the fluorescent channel corresponding to the fluorescence of Cy5.5 (excitation, 640 nm; emission, 647LP nm); middle: images in the fluorescent channel corresponding to the fluorescence of nuclear stain Hoechst3342 (excitation, 405 nm; emission, 445/45 nm); and right: merged images. Scale bar, 20 μm.

The MPQ-cytometry assay revealed that the conjugates of magnetic nanoparticles with affibody are more effective in terms of cell labeling (Figure 2c). To visualize this type of interaction, all of the conjugates were labeled with Cy5.5 fluorescent dye to get magnetic conjugates suitable for visualization in tissue transparency window. The excitation maximum of this fluorescent label is 683 nm, and the emission maximum is 703 nm. Cells were labeled with fluorescent magnetic conjugates and analyzed with two-channel confocal microscopy. The corresponding images are presented in Figure 3. The data presented confirmed that conjugates of magnetic particles with affibody Z_HER2:342_ are most efficient for cell labeling, in contrast to magnetic conjugates with DARPin and full-size IgG.

## Discussion

The development of nanoagents for the diagnostics and targeted therapy of cancer is one of the leading trends in modern biomedicine. Clinically significant oncomarkers that are overexpressed in certain types of malignant diseases, such as HER1, HER2, EpCAM, CD44, CD133, and others, are most often used as targets for drug delivery. In particular, here we use the HER2 cell surface receptor as a target for the delivery of nanoparticles due to a number of clinically relevant reasons. HER2 (also known as ErbB2, HER2/neu, neu) is a member of the epidermal growth factor receptor (EGFR) family. The *HER2* gene plays an important role in the formation and progression of malignant human tumors. It is amplified in ∼30% of human breast carcinomas and many other types of human malignant tumors, such as prostate carcinoma, endometrial cancer, primary gastric cancer, ovarian cancer, and kidney cancer. Overexpression of HER2 often correlates with patient resistance to chemotherapy, high tumor metastatic potential, and also predicts a high risk of disease recurrence and a decrease in overall patient survival. Thus, a highly selective influence on HER2-overexpressing cancer cells with HER2-directed compounds with diagnostic and therapeutic properties is of great clinical significance.

In this work, we have synthesized magnetic nanoparticles directed toward the receptor HER2. These particles are core–shell structures consisting of several magnetic cores of 23.3 ± 6.4 nm and coated with carboxymethyl-dextran (CMD). The stabilization of magnetic cores with CMD led to stability in saline solutions, thus making these particles suitable for biological applications. Moreover, this polymer has free −COOH groups available for further chemical modifications of the nanoparticle surface. Such nano-sized magnetic particles potentially have a good heat capacity and can be used to design magnetically induced local hyperthermia methods for cancer treatment.

Magnetic nanoparticles are one of the most common types of nanoparticles in biomedicine as agents for the therapy and diagnostics of a wide range of diseases. The diagnostic properties such as magnetic detection, MRI-contrast, the possibility of targeted delivery, and therapeutic properties such as magnetically induced hyperthermia and ferroptosis induction allow us to consider these particles as multifunctional theranostic agents.^21^ Traditionally, for the targeted delivery of magnetic nanoparticles to cells of interest *in vitro* and *in vivo*, full-length antibodies that recognize a specific molecular profile of cancer cells are used. Considering *in vivo* applications, such nanoparticles decorated with antibodies are far from ideal: the presence of a constant Fc domain of an antibody can lead to undesirable immunomodulation while significantly increasing the particle size. Moreover, in view of the biotechnological production of full-length antibodies, there are a number of significant reasons requiring the development of alternative delivery vehicles, such as complicated IgG folding, the presence of post-translational modifications, and protein production in expensive eukaryotic systems.

We have previously shown the effective delivery of DAPRin- and affibody-modified nanoparticles to cancer cells, such as anti-HER2 DARPin-liposomes,^22^ anti-HER2 DARPin-modified magnetic nanoparticles,^23^ affibody-decorated polymer particles,^24^ and DARPin-modified gold nanorods.^25^ But now there is no information on the direct comparison of these two classes of molecules for nanoparticle targeting. This work is a comprehensive study comparing the efficiency of the delivery of magnetic nanoparticles to HER2-overexpressing cells using scaffold proteins and full-length antibodies *in vitro*.

As-synthesized nanoparticles were successfully conjugated to HER2-recognizing ligands, namely, anti-HER2 full-size IgG trastuzumab, anti-HER2 DARPin_G3, and anti-HER2 affibody Z_HER2:342_ using carbodiimide chemistry. The synthesized nanoparticles were shown to successfully label the surface of HER2-positive cells.

Since HER2 is a receptor that is internalized and quickly recycled in the cell during conformational changes,^26^ it can be used as a mediator for the delivery of active compounds into the cell due to mainly clathrin-dependent endocytosis^27−29^ via all of the targeting ligands used in this study.

Indeed, it was originally shown that trastuzumab induces internalization and degradation of HER2.^30^ However, later studies concluded that trastuzumab alone induces internalization of HER2 only to a very limited extent,^31^ but when trastuzumab is cross-linked, e.g., using the biotin–avidin/streptavidin system, the internalization rate is quite high.^32^ Nanoparticles of different origins present such a multiple-ligand platform for the targeted delivery to cells. Along with full-size antibodies, affibody-conjugated^24,33−48^ and DARPin-conjugated^22,23,45,49^ magnetic nanoparticles were shown to be internalized into cells and localized in endosomes.

Using the original MPQ-cytometry method, we have shown that the efficiency of targeted delivery of magnetic nanoparticles using DARPin and affibody is higher than that using trastuzumab, a full-length anti-HER2 antibody with obvious superiority of affibody-modified particles. It should be also noted, however, that all of these proteins in the molecular form (labeled with FITC) demonstrate similar and efficient HER2-positive cell labeling under the flow cytometry assay. Since the size of affibody (8 kDa) is much smaller than those of IgG (150 kDa) and DAPRin (14 kDa), the most probable explanation of this phenomenon being the tight coverage of the nanoparticle surface with small affibody molecules in comparison to its counterparts. Moreover, since both N- and C-terms of affibody are far from the recognition site, the conjugation reaction does not significantly affect its recognizing properties.

By measuring the quantity of protein on the nanoparticle surface, we found that the number of DARPin molecules bound to the surface of nanoparticles is 10 times higher than the number of trastuzumab molecules, and the number of affibody molecules is 40 times higher than the number of trastuzumab molecules. The data obtained indicate that the smaller the molecule, the greater the amount of it that can be covalently attached to the nanoparticle surface, leading to the increase in the efficiency of targeted delivery (Figures 2 and 3).

Moreover, the higher level of binding of affibody-modified nanoparticles to cells can be explained by the higher avidity of such “nanoparticle–receptor” systems. The receptor HER2 in the cell membrane exists in the form of clusters and is associated with lipid rafts of about 67 nm consisting of about 9 receptors.^50^ If a nanoagent with a low ligand density is directed toward such a system, the binding is most likely described by a stoichiometric ratio equal to 1:1. If nanoparticles with a high ligand density are used, the binding is most likely to be multipoint, thus leading to a higher avidity of such interactions. It is important to note that this rafting effect is typical for various surface receptors, in particular tumor markers such as folate and transferrin receptors or ICAM-1.

Of course, this discussion is more applicable to uniform nanoparticles with the same number of ligands on the particle surface. However, almost every chemically synthesized nanoparticle has some degree of variability in size and surface properties that must be taken into consideration. To get the narrowest size distribution, one can vary the synthesis parameters, e.g., by separating magnetic fractions after the synthesis, and unify the number of ligands on the surface, e.g., by increasing the number of possible chemical grafting sites on the nanoparticle, or by using site-specific chemical reactions leading to 100% yield (e.g., click chemistry).

In particular, the as-synthesized nanoparticles possess quite high polydispersity, 264 ± 113 nm. Most likely, this is because the synthesized nanoparticles are multicore structures with a different number of magnetite cores inside one particle. At the same time, in this range of sizes of nanoparticles of different nature (150–400 nm), the effect of nonspecific binding with cells, caused by caveolin-mediated endocytosis, arises.^51^ The high nonspecific binding can be significantly reduced by conjugating recognizing molecules through the hydrophilic linkers (e.g., poly(ethylene glycol), PEG, or poly(ethylene oxide), PEO^52^), by varying the surface charge using other polymers instead of CMD (e.g., poly(lactic-*co*-glycolic acid), PLGA or poly(lactic acid), PLA^24^), or by using CMD in a combination with other polymers.

However, a higher number of ligands on the particle surface do not always mean better binding to cells.^39^ When a nanoparticle binds to a cell, there may be a problem associated with the very tight package of the recognition ligands on the nanoparticle surface. Too many recognition ligands, when placed very close to each other can lead to steric hindrance in target recognition or competition between two ligand molecules for one receptor molecule. Namely, it was previously shown^39^ that the ligand on the nanoagent surface significantly affects its ability to recognize the target, as well as to internalize into the cell. In particular, for a wide variety of targeting molecules (affibody or folic acid), it has been shown that there is a bell-shaped relationship between the number of surface ligands and their ability to specifically bind to target cells. In particular, for affibody molecules, this number was about 20 molecules per one superparamagnetic particle.

In this regard, due to the fact that at the same concentration it is possible to conjugate 40 times more affibody molecules compared to the number of full-size IgG, the much wider range of the number of ligands that can be conjugated to the surface becomes accessible below the surface saturation level of the nanoparticles. Thus, using affibodies for targeting, it is possible to fine-tune the properties of a nanoparticle for the required applications, for example, for labeling cells in immunoassays or for MRI-contrast enhancement. (It was previously shown that for the same particles the optimum number of molecules on the particle surface is different for different tasks.^39^)

## Conclusions

We showed that when conjugated to nanoparticles, small polypeptide scaffolds such as affibody are one of the most effective tools for the development of drug delivery systems. The synthesized magnetic conjugates are promising agents for diagnostic (MRI-contrasting) and therapeutic (local magnetically induced hyperthermia) applications with the obvious advantage of artificial polypeptide scaffolds over full-size antibodies.

## Materials and Methods

### Synthesis of Carboxymethyl-Dextran-Coated Magnetic Nanoparticles

Carboxymethyl-dextran-coated magnetic nanoparticles were synthesized by a microemulsion method. SDS (2 g) was dissolved in 20 mL of *n*-hexane and 12 mL of *n*-butanol using ultrasound until the mixture became transparent. The mixture was heated in a glass flask on a water bath for 20 min at 40 °C. FeCl_3_·6H_2_O (0.135 g) in 1 mL of Milli-Q water and FeSO_4_·7H_2_O (0.0834 g) in 0.6 mL of Milli-Q water were added to the emulsion to obtain the mixture with a light brown color. The temperature of the mixture was maintained at 40 °C for 20 min. Next, the heating temperature was increased to 70 °C and 2 mL of 25% NH_4_OH was added to obtain a black suspension. The resulting microemulsion was heated for 3 h at 70 °C and cooled to room temperature. Magnetic nanoparticles were magnetically separated and washed three times with 96% EtOH and three times with Milli-Q water. Next, the aggregates were magnetically removed, and nanoparticles in the supernatant were coated with carboxymethyl-dextran (CMD). For the coating reaction, a carboxymethyl-dextran solution at 300 g/L in Milli-Q water was added to the nanoparticles. Nanoparticles were incubated for 4 h at 90 °C. After that, the resulting nanoparticles were cooled for 5 min at 4 °C, after which they were heated again to 90 °C. This manipulation was repeated 3 times. Then, the resulting nanoparticles were washed from the nonbound carboxymethyl-dextran with Milli-Q water by triple magnetic separation.

### Dynamic Light Scattering (DLS) Measurements

The hydrodynamic size of magnetic nanoparticles and modified polymer-coated (CMD) nanoparticles were determined using the Zetasizer Nano ZS (Malvern Instruments) analyzer at a temperature of 25 °C in Milli-Q water or 1 M NaCl. Measurements were carried out in triplicate.

### Electron Microscopy

Scanning electron microscopy images of magnetite nanoparticles with a polymer coating (CMD) were obtained with a microscope MAIA3 Tescan (Tescan, Czech Republic). SEM images were evaluated using ImageJ software to achieve particle size distribution.

### SDS-PAGE

Scaffold proteins were produced in *E. coli* strain BL21 (DE3) according to the procedures described by us earlier for affibody Z_HER2:342_^24^ and DARPin_G3.^53^ Anti-HER2 trastuzumab was obtained from the medicine Herceptin (Roche) using the purification from stabilizing agents (l-histidine HCl, l-histidine, a,a-trehalose dihydrate, polysorbate 20) with NAP-5 size exclusion column according to the manufacturer’s recommendations. The concentration of as-obtained proteins was determined using the bicinchoninic acid (BCA) protein assay kit (Thermo) according to the manufacturer’s recommendations.

Protein identities were confirmed using sodium dodecyl sulfate-polyacrylamide gel electrophoresis (SDS-PAGE). Separating gel was prepared, applied to the glass sandwich, layered with isobutyl alcohol, and allowed to polymerize at room temperature. Then, the layer of isobutyl alcohol was poured off, and the gel was rinsed with H_2_O. The stacking gel was applied and allowed to polymerize at room temperature. Then, the comb was removed and the gel was rinsed and loaded to the Bio-Rad Mini-PROTEAN Tetra System chamber.

The sample (2–5 μg) was diluted with 2× sodium dodecyl sulfate (SDS)-containing sample buffer, heated for 5 min at 100 °C, and applied at the bottom of the wells as well as the Protein Ladder (Extra broad molecular weight, 5–245 kDa, ab116029, Abcam). The gel was run at 200 V until the tracking dye reached the bottom of the separating gel. The gel was stained for 15 min with Coomassie blue G-250 staining solution and washed with water or 10% acetic acid until the background became clear.

Trastuzumab was analyzed using 15% SDS-PAGE (Laemmli) gel consisting of 15% separating gel (15% acrylamide/0.4% bisacrylamide, 0.05% ammonium persulfate and 0.001% *N*,*N*,*N*′,*N*′-tetramethylethylenediamine (TEMED), 0.375 M Tris, 0.1% SDS, pH 8.8), 3.9% stacking gel (3.9% acrylamide/0.1% bisacrylamide, 25 μL of 0.05% ammonium persulfate and 0.001% TEMED, 0.1% SDS, 0.125 M Tris, pH 6.8), SDS electrophoresis buffer (0.25 M Tris base, 0.192 M glycine, 0.1% SDS), sample buffer (0.125 M Tris·Cl, pH 6.8, 20% glycerol, 4% SDS, 0.2% 2-mercaptoethanol, 0.001% bromophenol blue).

The scaffold polypeptides, DARPin_G3 and affibody Z_HER2:342_, were analyzed by SDS-PAGE (10%) using the Tris-tricine discontinuous electrophoresis system consisting of separating gel (9.8% acrylamide/0.26% bisacrylamide, 13% glycerol, 0.05% ammonium persulfate, 0.001% TEMED, 0.1% SDS, 1 M Tris, pH 8.45), stacking gel (3.9% acrylamide/0.1% bisacrylamide, 0.05% ammonium persulfate, 0.001% TEMED, 0.075% SDS, 0.75 M Tris, pH 8.45), anode buffer (0.2 M Tris base, pH 8.9), cathode buffer (0.1 M Tris base, 0.1 M tricine), and sample buffer (24% glycerol, 8% SDS, 0.02% Coomassie blue G-250, 0.2 M dithiothreitol (DTT), 0.1 M Tris·Cl, pH 6.8).

### Covalent Modification of Nanoparticles by Proteins

The obtained magnetic nanoparticles with polymer coating (CMD) were covalently modified with proteins (trastuzumab, DARPin_G3, affibody Z_HER2:342_, bovine serum albumin) using 1-ethyl-3-(3-dimethyl aminopropyl) carbodiimide, EDC, and N-hydroxysulfosuccinimide sulfo-NHS as zero-length cross-linking reagents. The nanoparticle modification reaction was carried out in two stages: first, the particles were activated with EDC/sulfo-NHS in 0.1 M morpholino ethanesulfonic acid (MES), pH 5.0, and then the excess of cross-linking reagents was removed using a magnetic separator. In the next stage, 70 μg of proteins in borate buffer (0.4 M H_3_BO_3_, 70 mM Na_2_B_4_O_7_·10H_2_O, pH 8.0) was added to nanoparticles. Nanoparticles with proteins were incubated at 4 °C for 12 h, after which the excess of unreacted substances was removed using a magnetic separator. The quantity of conjugated protein was measured by BCA protein assay kit (Thermo) according to the manufacturer’s recommendations.

Sulfo-cyanine5.5 NHS ester (Cy5.5)-labeled nanoparticle conjugates were prepared by mixing 100 μg (5 g/L) of magnetite nanoparticles with 1 μg of Cy5.5 in 10 μL of Milli-Q water. The reaction was carried out for 2 h, and then 50 μL of phosphate-buffered saline (PBS) with 1% bovine serum albumin (BSA) was added. The mixture was incubated for 12 h at 4 °C, after which the excess of unreacted Cy5.5 was washed with PBS seven times using a magnetic separator.

### Cell Culture

SK-BR-3 cells and CHO cells were cultured in Dulbecco’s modified Eagle’s medium (DMEM) supplemented with 10% heat-inactivated fetal bovine serum, 2 mM l-glutamine, and penicillin/streptomycin at 37 °C in a humidified atmosphere with 5% CO_2_. Cells were passaged two to three times a week at 80–90% confluency. The cells were removed from the plastic surface by 2 mM ethylenediaminetetraacetic acid (EDTA) in PBS, pH 7.4. Cell number counting was performed using an automatic Luna-II cell counter (Logos Biosystems, South Korea).

### MPQ-Cytometry

The number of nanoparticles bound to cells was quantified by MPQ-cytometry.^20^ The cells removed from the surface of the culture plastic were resuspended in PBS with 1% BSA at a concentration of 10^6^ cells/mL. A volume of 100 μL of cells was incubated with 7.5 μg of the studied conjugates for 30 min, and then unbound conjugates were washed three times with centrifugation at 80 g. The number of magnetic nanoparticles bound to cells was quantified with the MPQ device.

### Flow Cytometry

FITC-labeled proteins were prepared as described by us earlier.^24^ FITC-labeled protein (2 μg) was mixed with 300 × 10^3^ cells in 300 μL of PBS with 1% BSA. Cells were incubated for 30 min, washed from nonbound substances with triple centrifugation, and analyzed with flow cytometry using BD Accuri C6 (BD) device in FL1 channel (excitation, 488 nm; emission, 525/25 nm). A total of 10 000 singlet cells were collected. Data were analyzed with CFlow Plus and FlowJo software.

### Cytotoxicity Assay

The cytotoxicity of the synthesized targeted nanoparticles was investigated using a resazurin toxicity assay. Cells were seeded on a 96-well plate at 2 × 10^3^ cells per well in 100 μL of DMEM supplemented with 10% FBS, cultured overnight, and then nanoparticles were added. The cells were cultured for 3 days. Next, 100 μL of resazurin solution (13 mg/L in PBS) was added to each well, and the samples were incubated for 2 h at 37 °C in a humidified atmosphere with 5% CO_2_. The fluorescence of each well was measured using Infinite 1000 Pro (Tecan, Austria) microplate reader at wavelengths of λ_ex_ = 570 nm and λ_em_ = 600 nm. Data are presented as percent from nontreated cells.

### Confocal Microscopy

For confocal microscopy analysis, the cells were incubated with Cy5.5-labeled nanoparticles as for MPQ-cytometry assay, resuspended in PBS with 1% BSA, and seeded into flat-bottom 96-well glass plates at a concentration of 2 × 10^4^ cells/mL. The cells were analyzed with confocal laser scanning microscopy using a Leica DMI6000B (Leica Microsystems, Germany) microscope equipped with Confocal Microscopy Upgrade (Thorlabs) for Hoechst 33342 imaging: excitation laser – 405 nm, emission filter – 445/45 nm; for Cy5.5 imaging: excitation laser – 640 nm, emission filter – 647LP nm.
